# Does the Interaction between Local and Systemic Inflammation Provide a Link from Psychology and Lifestyle to Tissue Health in Musculoskeletal Conditions?

**DOI:** 10.3390/ijms22147299

**Published:** 2021-07-07

**Authors:** David M. Klyne, Mary F. Barbe, Greg James, Paul W. Hodges

**Affiliations:** 1 NHMRC Centre of Clinical Research Excellence in Spinal Pain, Injury and Health, School of Health and Rehabilitation Sciences, The University of Queensland, Brisbane 4072, Australia; g.james1@uq.edu.au (G.J.); p.hodges@uq.edu.au (P.W.H.); 2 Department of Anatomy and Cell Biology, Lewis Katz School of Medicine, Temple University, Philadelphia, PA 19140, USA; mary.barbe@temple.edu

**Keywords:** fibrosis, local inflammation, systemic inflammation, psychological factors, lifestyle factors

## Abstract

Musculoskeletal conditions are known to involve biological, psychological, social and, often, lifestyle elements. However, these domains are generally considered in isolation from each other. This siloed approach is unlikely to be adequate to understand the complexity of these conditions and likely explains a major component of the disappointing effects of treatment. This paper presents a hypothesis that aims to provide a foundation to understand the interaction and integration between these domains. We propose a hypothesis that provides a plausible link between psychology and lifestyle factors with tissue level effects (such as connective tissue dysregulation/accumulation) in musculoskeletal conditions that is founded on understanding the molecular basis for interaction between systemic and local inflammation. The hypothesis provides plausible and testable links between mind and body, for which empirical evidence can be found for many aspects. We present this hypothesis from the perspective of connective tissue biology and pathology (fibrosis), the role of inflammation locally (tissue level), and how this inflammation is shaped by systemic inflammation through bidirectional pathways, and various psychological and lifestyle factors via their influence on systemic inflammation. This hypothesis provides a foundation for new consideration of the development and refinement of personalized multidimensional treatments for individuals with musculoskeletal conditions.

## 1. Introduction

**Figure 1:**
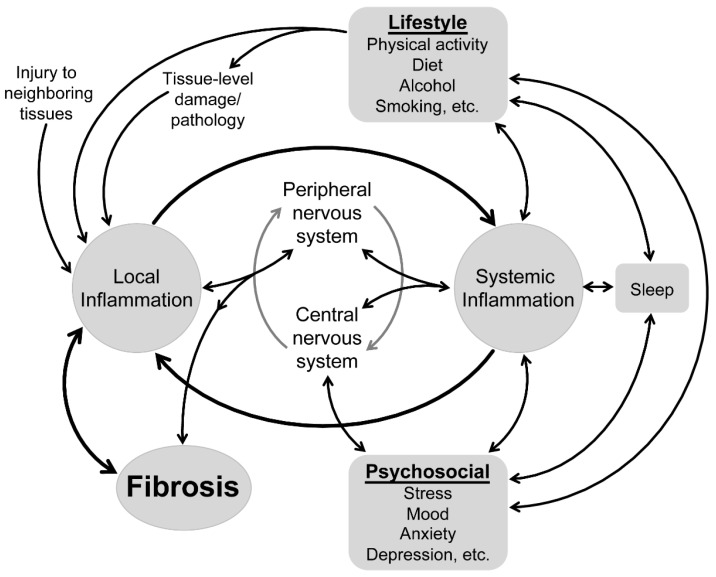
Conceptual model of the factors that impact tissue health in musculoskeletal conditions.

Changes in connective tissue influence the physiological and mechanical functioning of musculoskeletal tissues and are increasingly recognized in musculoskeletal conditions [1]. Understanding of the physiology and pathophysiology of musculoskeletal connective tissue in health and disease has traditionally focused on local “within-muscle” processes, including the inflammatory responses that actively regulate connective tissues. Looking beyond the local tissues, parallel research is beginning to reveal a potential role for systemic inflammatory changes in shaping these local inflammatory processes and, thus, connective tissues. As systemic inflammation is sensitive to a range of psychosocial and lifestyle factors, this new work provides a foundation to begin to consider how these factors might impact connective tissue health via their interaction with the systemic then local inflammatory systems (Figure 1). This review presents, from a molecular point of view, a hypothesis that is derived from a series of recent findings that has the potential to explain a novel link between “body and mind” in musculoskeletal conditions. Although proof of this link is not yet available, we present a series of testable questions with the objective to open new research directions to further understand the complexity of musculoskeletal health.

## 2. The Role of Local Inflammation in Musculoskeletal Connective Tissue Health

Tissues, including connective tissues, generate an inflammatory response in multiple contexts. This includes the response that is essential for the clearance of necrotic cells and repair after tissue injury [2,3,4], exercise [5] and the disruption of related structures, such as can occur to muscles that cross an injured joint [6]. Under normal conditions, this inflammation initiates mechanisms to drive adaptation and repair, including amplification of the inflammatory response as needed by activating soluble mediators and immune cell infiltration. This response is eventually terminated by specific inhibitors and followed by regenerative processes to restore normal tissue architecture and function. Under certain conditions, the ability to clear injured tissues is impaired (e.g., as with diabetes or aging), regulatory mechanisms may be altered, local inflammation becomes persistent and systemic or injury-repair cycles are too persistent for the complete resolution of inflammation. To understand the impact of this process on musculoskeletal connective tissue, it is first critical to understand its relevant anatomy and how local tissue inflammation is regulated.

### 2.1. Overview of Musculoskeletal Connective Tissue

Skeletal muscles, tendons and nerves are each hierarchically organized tissues that employ structural bundling. The individual elements—myofibers in muscles, collagen fibrils in tendons and axons and Schwann cell sheaths in nerves—are surrounded by a small amount of delicate connective tissue (endomysium, endotenon or endoneurium, respectively), which are bundled into fascicles by a slightly denser collagenous connective tissue (perimysium, peritenon or perineurium). The entire muscle, tendon or nerve is surrounded by a dense external sheath of connective tissue (epimysium, epitenon or epineurium). These fascial components act as cushions, allow movement and transfer of force between the tissues and carry blood vessels and nerves into and out of subcomponents [7,8]. Numerous cell types are found within these connective tissues, including fibroblasts, adipocytes, lymph vasculature cells and various migrating immune cells. Fibroblasts, a type of mesenchymal cell, are the primary support cells. They are heavily involved in the turnover of extracellular matrix (ECM) and are key producers of collagen, especially after differentiating into myofibroblasts in response to injury or growth factor exposure, e.g., transforming growth factor beta (TGF-β1). During stress, fibroblasts adapt to their environment and can respond to and send local signals, including inflammatory mediators [9]. This review is primarily focused on connective tissue in and around muscles.

### 2.2. Injury-Induced Acute Inflammation and Normal Tissue Repair

Acute inflammation is characterized by a rapid accumulation of fluid, neutrophils and plasma cells in the affected tissue. The first signals released from damaged and necrotic cells are damage-associated molecular pattern molecules (DAMPS) that include interleukin-1 alpha (IL-1α; proinflammatory cytokine). DAMPs are chemotactic for neutrophils, as are several chemokines (chemotactic cytokines) produced by neighboring uninjured cells after DAMP exposure [3]. The neutrophil influx is rapid and typically short-lived (2 h) [3,10]. Functionally, neutrophils adopt three possible strategies: (1) the removal of cellular debris by phagocytosis, an activity shared with macrophages that are also attracted by chemotactic factors, (2) the release of growth and angiogenic factors and/or (3) the resolution of inflammation [3]. The latter occurs when neutrophils become apoptotic and are phagocytosed by macrophages, a process that initiates a feed-forward pro-resolution program characterized by the release of tissue repair-related cytokines, e.g., TGF-β1, and the potent anti-inflammatory cytokine, interleukin-10 (IL-10). Thereafter, neutrophils rapidly decline and are replaced by macrophages [11,12,13,14].

Macrophages are activated and polarized into M1 (proinflammatory) or M2 (anti-inflammatory, repair or both) phenotypes [12,15]. Many M1 macrophages are bone marrow-derived phagocytes recruited to the injured site by chemotactic and adhesion molecules and proinflammatory cytokines [16]. M1 macrophages are both activated by and produce proinflammatory cytokines, including IL-1α and β and tumor necrosis factor (TNF) [17]. Their influx is required for the clearance of injury-induced debris [18,19,20].

The next essential step for tissue repair and regeneration is a transition from a proinflammatory milieu to an anti-inflammatory one [19]. Increases in anti-inflammatory cytokines, such as IL-10 and interleukin-13 (IL-13), inhibit most proinflammatory responses and stimulate increases in repair-related M2 macrophages [21]. The functional subtypes of M2 macrophages include: M2a (anti-inflammatory macrophages that promote muscle wound healing or fibrosis in response to interleukin-4 (IL-4) and IL-13), M2b (macrophages that activate in response to immune complexes and lipopolysaccharide (LPS)) and M2c (deactivated macrophages that respond to IL-10, TGF-β1 and glucocorticoids to then secrete IL-10 and TGF-β1, furthering tissue repair) [18].

This predominantly cytokine-driven acute phase response occurs not only at the localized site of injury for immune cell recruitment but, also, in distant tissues, including the liver and brain, in order to amplify or depress humoral defense components [22,23]. The acute phase response involves a complex array of neuroendocrine, physiological and metabolic changes initiated both locally and systemically after tissue infection or injury, directly or secondary to the initial insult or other assaults to the body, such as muscle damage in response to exercise [5]. These latter two scenarios are discussed below.

### 2.3. Inflammation Induced by Injury to Adjacent Structures

Local muscle inflammatory responses and connective tissue adaptation is initiated not only by direct injury to muscle but, also, in response to injuries to related (nearby) joint structures. This has emerged as an explanation for structural changes in the paraspinal muscles (particularly the multifidus muscle) that are commonly observed in low back pain [24,25], and in animal models of intervertebral disc lesion [26]. In addition to muscle atrophy and fat accumulation, these muscles undergo extensive connective tissue adaptation, including thickening of the epimysium and changes in the collagen subtype [27]. Consistent with the processes highlighted above to explain the accumulation of connective tissue after muscle injury, animal models show that the development of a degenerative cascade in the intervertebral tissues (either following injury [26] or with spontaneous disc disease [27,28]) is associated with an inflammatory response in adjacent paraspinal muscles, as evidenced by the increased expression of proinflammatory cytokines (e.g., TNF and IL-1β [6]), fibrogenic molecules (e.g., CCN2 [25,27]) and the accumulation of macrophages [29] and their polarization to the proinflammatory M1 type [30]. Notably, TNF levels first increase in the muscle but are then amplified in the connective tissues [30]. This inflammatory response does not begin immediately after injury, and, in the absence of injury to the muscle, the primary role is unlikely to be one of removal of the cellular debris. Instead, this response appears to serve a role in adaptation of muscle structure, such as connective tissue remodeling. These changes are partially, but not completely, resolved/reversed by exercise [27].

### 2.4. Exercise-Induced Inflammation in Muscles

Inflammatory cytokines are released from contracting skeletal muscle cells during exercise [5]. IL-6 is the most predominant cytokine produced by exercising muscles, and is the first to increase in circulation, with levels peaking immediately post-exercise before rapidly declining to baseline concentrations [31]. For instance, prolonged strenuous running results in elevated systemic levels of IL-6, as well as counteracting anti-inflammatory mediators such as IL-10 and interleukin-1 receptor antagonist (IL-1Ra), which peak within 0–90 min and typically resolve within ~6 h, post-exercise [32,33]. Similar cytokine temporal patterns occur for other exercise types, but the magnitude by which IL-6 increases is highly dependent on the exercise intensity and duration, and muscle mass involved [34]. Muscle-derived IL-6 is produced in response to declining glycogen levels, independent of muscle damage [5]. Once in circulation, IL-6 signals the liver to produce glucose and adipocytes to produce free fatty acids (i.e., lipolysis) needed for muscle energy. IL-6 also acts as a paracrine growth factor, promoting signaling for satellite cells during regenerative myogenesis [35]. Thus, metabolic and tissue remodeling functions require elements of inflammation.

Although skeletal muscle damage is not required for the release of IL-6 from muscle during and after exercise, prolonged elevated concentrations of muscle-derived IL-6 are thought to indicate greater damage resulting from microtrauma to the contractile elements and connective tissue within muscle [31,36]. Under these conditions, immune cells infiltrate the muscle and secrete cytokines, including IL-6, for an extended period of time to repair the damaged tissue [5,31,36,37]. Over time, as the skeletal muscle adapts with training, the IL-6 response to exercise is blunted. However, repeated exercise bouts with limited recovery between repetitions can have the opposite effect with untoward physiological effects [36], similar to those observed in repetitive overuse injuries (see Section 2.5 and Section 3.2).

### 2.5. Chronic Inflammation and Fibrotic Muscle Repair

Under some conditions, the ability to clear injured tissues of pathogens and debris is impaired, mechanisms regulating inflammatory responses are altered and/or injury–repair cycles are too persistent for the complete resolution of inflammation. In either case, inflammation can become enhanced, persist and spread to include previously uninjured tissues. Inflammatory cytokines in tissues can also elevate to detectable levels in circulation, a change that is often paralleled by inflammatory responses at sites distant from the initial injury site [38]. Unresolved or adherent systemic inflammatory responses may influence the inflammatory environment in otherwise healthy, uninjured tissues, including muscles.

Chronic inflammation is characterized by the prolonged presence of inflammatory M1 macrophages and neutrophils that have amplified the primary acute inflammatory response by their enhanced production of proinflammatory cytokines. Their continued phagocytosis and release of cytotoxic effectors (e.g., cytokines, reactive oxygen/nitrogen species and proteolytic enzymes) promotes secondary tissue damage in cells and tissues surrounding the original injury site [18]. The experimental depletion of neutrophils or blocking their function reduces this collateral damage but also reduces tissue repair and regeneration [2,39]. Similarly, the experimental depletion of macrophages early post-injury diminishes the inflammatory response but also decreases wound debridement, tissue repair and regeneration [40,41]. These findings highlight the complex nature of these biological processes and the limitations of pharmacotherapies targeting immune cells.

Repeated injury and damage cycles in muscles are associated with marked increases in noncontractile tissues (primarily collagen) [42,43]. This may be due to the prolonged presence and activity of M2a type macrophages that drive fibroblast proliferation and collagen production [11,12,13]. Increased TGF-β1 also promotes fibroblast proliferation and collagen production, plus the differentiation of fibroblasts into myofibroblasts [44]. Myofibroblasts have high levels of actin and are contractile; however, they are also heavy producers of collagen and contribute to excessive collagen deposition (i.e., fibrosis) with prolonged activation [45]. TGF-β1 also potently induces other fibrogenic proteins, including CCN2 (cellular communication network factor 2; also known as connective tissue growth factor (CTGF)) [46,47]. CCN2 is a secreted matricellular protein with four modular domains that independently interact with and signal numerous molecules, such as collagen and proteoglycans in the ECM [48], and plays a key role in ECM overproduction and tissue fibrosis [49]. The pharmacological blocking of CCN2 signaling reduces macrophage numbers, macrophage-related gene signatures and chemokine mRNA production in animal models of tissue damage [50,51,52]–suggesting that CCN2 also modulates inflammation. Similarly, the attenuation of CCN2 expression by aerobic exercise in a model of low back pain was mirrored by attenuated local inflammatory and fibrotic responses in muscle [27]. Not surprisingly, CCN2 is markedly elevated in serum and fibrotic organs/tissues collected from animals and humans with fibrotic conditions, including muscular dystrophy and repetitive overuse injury [50,53,54,55]. Its systemic presence may indicate a contribution to the pathogenesis of widespread tissue fibrosis.

## 3. The Role of Systemic Inflammation in Musculoskeletal Connective Tissue Health

The notion that inflammatory events can impact remote tissue health has been identified in some chronic conditions (e.g., diabetes) but has been relatively unexplored in musculoskeletal conditions. The overall concept is underpinned by the dynamic feedback loops between local tissue and systemic inflammation. Elevated systemic inflammation induces a proinflammatory response in tissues, such as muscles distant from an injury site [56]. Conversely, inflamed tissues secrete cytokines (such as myokines from muscles) that contribute to systemic inflammation [57]. This self-perpetuating cycle could underpin a chronically elevated inflammatory state [58,59] with profound effects on the physiology and structure of musculoskeletal tissues, including connective tissues. Supporting this supposition is the association between chronically high levels of circulating cytokines and low muscle mass and strength [60]. Systemic inflammation is a key component of various conditions that involve tissue fibrosis, such as obesity, diabetes and cachexia [61,62,63,64,65], and has begun to be identified in some chronic musculoskeletal conditions, such as low back pain [66,67,68,69]. The following highlights the potential physiology underlying the impact of systemic inflammation on local inflammation related to muscles and connective tissues.

### 3.1. The Interplay between Systemic and Local Inflammation in Musculoskeletal Tissues

Changes in systemic inflammation alter the inflammatory environment in musculoskeletal tissues. The induction of acute systemic inflammation via intraperitoneal injection of LPS in animals increases the expression of TNF, IL-6 and IL-1β in skeletal muscle within 2–12 h [70,71]. Moreover, it drives increased expression of genes associated with M1 macrophages and reduced expression of genes associated with M2 macrophages, suggesting a role in initiating the proinflammatory polarization of macrophages [70,71]. Other examples include the increased secretion of IL-6 from muscles in humans after acute systemic infusion of TNF [72] and enhanced systemic inflammation and TNF expression in muscles following experimentally induced TNF overexpression in lung tissue [73]. These “systemic-to-local” effects are thought to be regulated by Toll-like receptor 4 (TLR4), which activates the innate immune system [72]. Pharmaceutical blockage of TLR4 activation or its genetic ablation attenuates systemically driven local inflammatory responses (e.g., proinflammatory cytokine expression) and induces a shift toward “anti-inflammatory” M2 macrophages [74,75,76]. These findings are mirrored by reduced muscle fibrosis [74]. These and other examples [56] highlight the potential for systemic inflammation to mediate local inflammation and enhanced connective tissue deposition.

The impact of “chronically” elevated systemic inflammation on local tissue inflammation is complex and difficult to directly assess. Insights can be drawn from studies that have examined muscles from patients with conditions characterized by chronic low-grade systemic inflammation, such as obesity, diabetes and some chronic musculoskeletal conditions (Table 1). Muscle biopsies from humans with these conditions and animal models show infiltration of macrophages and neutrophils, M1 macrophage polarization and increased expression of proinflammatory cytokines and other mediators (including TLR4) [28,30,77,78]. Systemic anti-inflammatory treatments attenuate macrophage infiltration, M1 polarization and proinflammatory cytokine/mediator expression in these conditions and promote the expression of anti-inflammatory cytokines [79,80].

### 3.2. The Role of Systemic Inflammation in Regulating Fibrogenesis

The impact of systemic inflammation on the local inflammatory environment, and their bidirectional relationship, provides a potential explanation for enhanced fibrosis in musculoskeletal tissues. Elevated levels of circulating proinflammatory cytokines are simultaneously present with fibrosis in a wide range of conditions [59]. Chronic muscle and tendon overuse induces increases in systemic levels of TNF and IL-6 and the fibrogenic proteins TGF-β1 and CCN2 in musculoskeletal tissues [81,82,83]. At the tissue level, numerous inflammatory and fibrotic alterations have been observed not only within the directly affected and neighboring muscle(s) but, also, distant, unrelated muscles [38,55,82,83,84,85,86], highlighting the potential influence of systemic mediators. Individuals with chronic low back pain present with both elevated circulating TNF [66,67,68,69] and fibrotic activity in paraspinal muscles, including increased CCN2 expression and connective tissue accumulation [25]. CCN2 (which is regulated by TNF [87]) is even known to synergize with IL-1β to amplify inflammatory responses, e.g., enhanced IL-6 production and release [88,89]. Similar inflammatory and fibrotic profiles are observed in other musculoskeletal conditions (Table 1). Non-musculoskeletal conditions that present with systemic and remote local inflammatory changes (as suggested above) are also characterized by fibrotic changes (Table 1). Despite differences in the etiologies between these musculoskeletal and non-musculoskeletal conditions, the inflammatory pathways appear similar, with elevated expression of circulating cytokines, primarily TNF, IL-1β and IL-6 (Table 1). In conditions such as diabetes, this is likely to underpin fibrosis in remote tissues such as the retinas and kidneys in a similar manner to that currently proposed in musculoskeletal tissues. The mechanisms mediating this interaction are beginning to be understood.

The Autotaxin/Lysophosphatidic acid (ATX/LPA) pathway mediates inflammation and fibrosis and is upregulated in numerous fibrotic conditions (e.g., idiopathic pulmonary fibrosis, scleroderma and renal interstitial fibrosis) [90]. LPA is an active glycerophospholipid that upregulates local proinflammatory and fibrotic responses in muscles, providing a plausible link between circulating inflammatory cytokines and fibrotic activity in muscles [91,92,93]. Systemic TNF is a potent promotor of ATX expression, which synthesizes LPA. In a model of muscle injury, the intraperitoneal administration of LPA increased the infiltration of neutrophils and macrophages into the muscle and upregulated muscle TNF and TGF-β1 expression and, ultimately, muscular fibrosis [91]. Current evidence suggests that LPA promotes muscle fibrosis via the induction of CCN2 expression [93]. Other pathways are also possible. Further examination of these pathways in models of chronic inflammation is required.

**Table 1:** Examples of musculoskeletal and chronic metabolic/lifestyle-related conditions characterized by inflammation and fibrosis at both the systemic and muscle levels.

Condition	Systemic Inflammation/Fibrotic Proteins	Muscle Inflammation	Muscle Fibrosis
**Musculoskeletal/neuromuscular conditions**			
Complex regional pain syndrome	<6 months after symptoms onset: - ↑ IL-8, sTNFR1/2 [94] >6 months after symptoms onset: - ↑ CCL2, IL-2, IL-4, IL-7, IL-1Ra, IFNγ, sIL-1R1, sIL-2Ra, sRAGE, TNF - ↓ SP [94]	≤4 weeks after initiation in animal model; local and/or nearby muscles (lower limb): - ↑ IL-1β, IL-6, MDA, NF-κB, TNF [95,96,97]	Chronic phase; local and nearby muscles (upper and lower limbs): - ↑ connective tissue [98] 2 weeks after initiation in animal model; local and nearby muscle (lower limb): - ↑ TGF-β1 - Thickened epimysium, perimysium, endomysium [96]
Low back pain	Acute phase (<2 weeks): - ↑ CRP, IL-6, TNF [66,67] Late/chronic-phase (>3 months): - ↑ TNF [67,69]	Late/chronic phase (>6 months); nearby muscle (multifidus): - ↑ IL-1β, TNF - M1 macrophage polarization [6,28]	Late/chronic phase (>6 months); nearby muscle (multifidus): - ↑ CCN2, TGF-β1, connective tissue (including Collagen-I/III, fibronectin) - Thickened epimysium, proliferation of FAPs [25,27]
Rheumatoid arthritis	Early/late disease stage: - ↑ CRP, IL-1β, IL-6, TNF - Cytokines correlate with disease severity and activity [99,100,101,102]	Late/chronic disease stage; nearby muscles (lower limb): - ↑ IL-1β, IL-6, IL-8, TNF - M1 macrophage polarization [77,99,103]	Late/chronic disease stage; nearby muscles (lower limb): - ↑ collagen, amino acid precursors to fibrosis [77,99,103]
Work-related overuse injury/repetitive strain injury	Early/acute phase (≤3 weeks): - ↑ CXCL10, IL-1β [82] Sub-chronic phase (6–12 weeks): - ↑ CCL20, CXCL2, CRP, IL-1α/β, IL-6, TNF [38,58,104,105] Late/chronic phase (>12 weeks): - ↑ CCN2, collagen, markers of collagen metabolism, CRP, IFNγ, IL-1α, IL-6, IL-10, IL-12, sIL-1R, TGF-β1, TNF, TNFR1, visfatin [55,82,83,84,106,107,108,109,110]	Early/acute phase; local and nearby muscles: - ↑ CCL2, IL-1α/β, IL-18, glutamate, pyruvate, TNF - M1 macrophage polarization [38,50,81,82,83,111,112,113] Subchronic phase; local, nearby and/or distant muscles: - ↑ IL-1α, IL-1β (muscles generally), IL10, TNF - M1 (distant muscles only) and M2 macrophage polarization [38,58,81,85] Late/chronic phase; nearby and distant muscles: - ↑ IL-6, IL-10, TNF [83]	Early/acute phase; local and nearby muscles: - ↑ myofibroblasts, Collagen-I, TGF-β1 [50] Subchronic phase; local and nearby muscles: - ↑ CCN2, Collagen-I, TGF-β1 [79,82] Late/chronic phase; local, nearby and/or distant muscles: - ↑ CCN2, Collagen-I/III, FGF2, IFNγ, PDGF, TGF-β1 - Persistent M2 fibrogenic CD20+ macrophages [55,82,83,84,86]
Duchenne muscular dystrophy	Early/late disease stage: - ↑ IL-6, sRANKL, TNF [114,115,116]	Early/late disease stage; various muscles: - ↑ CCL2/7/8, IFNγ, IL-1β, IL-4, IL-5, IL-8, IL-17, M1 macrophages, MIP-1, NF-κB, TNF - ↓ M2 macrophages [115,116,117,118]	Early/late disease stage; various muscles: - Plethora of profibrotic alterations, e.g., ↑ CCN2, TGF-β1/β3, collagen (types I, III, IV, V, VI, XIV, XV, XVIII), connective tissue, ECM gene dysregulation, etc. [115,116,119]
**Chronic metabolic/lifestyle-related conditions**			
Alcoholism/alcoholic myopathy	- ↑ CCL2, CRP, IL-1α/β, IL-6, IL-8, IL-12, IL-15, TNF, MDA [120,121,122]	- ↑ IL-6, ROS, TNF [123,124,125]	- ↑ TGF-β1, collagen - Altered expression of genes involved in ECM [123,126,127]
Cancer related Cachexia	- ↑ IL-1β, IL-6, TGF-β1, TNF [62,128]	- M1 macrophage polarization [129]	- ↑ CCN2, collagen - Transformation of fibroblasts into myofibroblasts [62]
Chronic Obstructive Pulmonary Disease	- ↑ CRP, IL-1β, IL-6, IL-8, TNF [73,130,131]	- ↑ IL-6, TNF [73,131]	- ↑ collagen [132]
Diabetes	- ↑ IL-1, IL-6, IL-10, leptin, TNF [65,130]	- ↑ IL-6, IL-10, TNF - Macrophage accumulation [133]	- ↑ in ECM components: Collagen -I/III/IV, fibronectin, integrin [61]
Obesity	- ↑ CRP, IL-1, IL-6, IL-8, leptin, resistin, TNF, visfatin [134] - ↓ adiponectin [63,64,135]	- ↑ IL-8 and TNF - M1 Macrophage polarization	- ↑ collagen in ECM - Proliferation of FAPs [63]

Abbreviations: CCL2—chemokine (C-C motif) ligand 2; CCL7—chemokine (C-C motif) ligand 7; CCL8—chemokine (C-C motif) ligand 8; CCL20—chemokine (C-C motif) ligand 20; CCN2—cellular communication network factor 2; CRP—C-reactive protein; CXCL2—chemokine (C-X-C motif) ligand 2; CXCL10—C-X-C motif chemokine ligand 10; ECM—extracellular matrix; FAPs—fibro/adipogenic progenitors; FGF2—fibroblast growth factor 2; IFNγ—interferon gamma; IL-1α—interleukin-1 alpha; IL-1β—interleukin-1 beta; IL-2—interleukin-2; IL-4—interleukin-4; IL-5—interleukin-5; IL-6—interleukin-6; IL-7—interleukin-7; IL-8—interleukin-8; IL-10—interleukin-10; IL-12—interleukin-12; IL-15—interleukin-15; IL-17—interleukin-17; IL-18—interleukin-18; IL-1Ra—interleukin-1 receptor antagonist; MDA—malondialdehyde; MIP-1—macrophage inflammatory protein-1; NF-κB—nuclear factor kappa-light-chain-enhancer of activated B cells; PDGF—platelet-derived growth factor; ROS—reactive oxygen species; sIL-1R1—soluble interleukin-1 receptor type 1; sIL-2Rα—soluble interleukin-2 receptor alpha; SP—substance P; sRAGE—soluble receptor for advanced glycation end-products; sRANKL—soluble receptor activator of nuclear factor (NF)-κB ligand; sTNFR1—soluble tumor necrosis factor receptor 1; sTNFR2—soluble tumor necrosis factor receptor 2; TGF-β1—transforming growth factor beta 1; TGF-β3—transforming growth factor beta 3; TNF—tumor necrosis factor; TNFR1—tumor necrosis factor receptor 1.

## 4. Does the Impact of Systemic Inflammation on Local Inflammation Provide a Link between Psychological and Lifestyle Factors in Health of Musculoskeletal Connective Tissues?

The systemic inflammatory environment is modulated by factors other than those directly associated with “immune” function. These include psychological or lifestyle features, and the health consequences are broad. Growing evidence of the capacity for systemic inflammation to influence local inflammation provides a plausible link between these factors and the health of musculoskeletal tissues.

### 4.1. Links between Psychological Factors and Systemic Inflammation

Many psychosocial factors are associated with increased systemic levels of proinflammatory mediators (e.g., TNF, IL-1β and IL-6) and decreased anti-inflammatory mediators (e.g., IL-10) [67,136,137,138,139]. Meta-analyses suggest these factors can trigger a systemic inflammatory response by disrupting the balance of the immune system [140,141,142]. Acute psychosocial stressors (e.g., work, family and financial stress) activate the sympathetic nervous system (SNS) and hypothalamic pituitary adrenal axis (HPA axis), which are together responsible for the maintenance or reinstatement of homeostasis during stress [143]. The SNS promotes the secretion of catecholamines (i.e., dopamine, epinephrine and norepinephrine) from the adrenal medulla and sympathetic nerve endings via several mechanisms (for review, see references [143,144]). Once released, they stimulate the upregulation of proinflammatory cytokines, which increases systemic inflammation [139,145]. This is followed by slower activation of the HPA axis—hypothalamic secretion of corticotropin-releasing hormone (CRH), which triggers the pituitary secretion of adrenocorticotropic hormone (ACTH), which then stimulates the release of glucocorticoids (cortisol) by the adrenal glands [143]. Both catecholamines (proinflammatory) and cortisol (anti-inflammatory) regulate inflammation and, under normal circumstances, work synchronously to restore internal homeostasis and turn off the stress response by negative feedback inhibition [143]. A different set of dynamics can emerge under conditions of recurring or sustained stress, leading to HPA axis-related increases (rather than decreases) in inflammation [139]. This phenomenon is related, at least in part, to reduced feedback inhibition by glucocorticoids (i.e., cortisol). Through binding to their receptors (glucocorticoid receptor) in the HPA axis, glucocorticoids are potent negative regulators of the HPA axis [143]. When glucocorticoid levels persist, their receptors can become less sensitive, altering negative feedback regulation and leading to excessive glucocorticoid secretion [144,146]. Similarly, immune cells become less sensitive to the anti-inflammatory effects of glucocorticoids in order to compensate for their persistence [146,147]. With cortisol unable to suppress inflammation, stress continues to promote proinflammatory cytokine production (via the SNS) indefinitely.

Consistent with the capacity for stress to modulate immune function, and the role of psychosocial stress as a risk factor for the development of various psychological conditions [148,149], inflammatory processes are considered central to the pathogenesis of conditions such as depression and anxiety [150,151,152]. For instance, peripherally released cytokines can access the brain by crossing the blood–brain barrier at specific sites (e.g., circumventricular organs) or send signals via TLRs that stimulate cytokine expression in the brain [153,154,155,156]. Once in the brain, there is a cytokine network consisting of neural and glial elements that not only produce cytokines and express cytokine receptors but also amplify cytokine signals, all of which can profoundly affect neurotransmitters (serotonin, norepinephrine and dopamine) that impact psychological conditions (and mood regulation), pain and other behaviors [150,157]. In turn, stress is likely to perpetuate and continue to trigger systemic inflammation.

Although very few, if any, studies have focused on the potential implications of inflammation induced by stress/psychology on the health of tissues, the notion is supported by empirical evidence. For example, stress is associated with inflammatory-related symptoms (e.g., swelling) in rheumatoid arthritis [158,159,160]. Stress is also a risk factor for the development of persistent pain [161,162] and inflammatory-driven musculoskeletal diseases [163,164], and is associated with disease exacerbation [165,166]. A key consideration in these contexts is that stress/psychological factors and inflammation are bidirectionally linked—one can mediate the other via the HPA axis. Animal studies have shown that peripheral inflammatory events in the absence of stress, such as those that occur after tissue injury, induce psychological and behavioral symptoms [167,168,169,170]. Regardless of the direction of causality, persistent psychogenic stress and unmoderated inflammation could have a synergizing effect on the HPA axis, amplifying the cycle of psychological distress, systemic and local inflammation, and tissue pathology.

### 4.2. Links between Lifestyle Factors and Systemic Inflammation

Research is continuing to reveal prominent candidate factors that induce systemic inflammation. Sleep is a well-known modulator of systemic inflammation [171,172]. Sleep aids in the recovery from infections and other disease states by regulating major effector systems of the CNS such as the HPA axis and the SNS (like stress), as well as the immune system [173]. The different stages of sleep (i.e., rapid eye movement sleep and the three stages of non-rapid eye-movement sleep) differently regulate nocturnal levels of inflammatory cytokines, and this lays the foundation for inflammatory levels throughout the day [174,175,176,177]. Sleep loss or disturbance can alter this inflammatory balance (via activating the stress response [178] and/or inflammatory signaling pathways, e.g., those involving nuclear factor-κB (NF-κB), activation protein-1 (AP-1) and signal transducer and activator of transcription (STAT) family proteins [172,179,180,181]), initially inducing a shift in the temporal pattern of inflammatory responses, with increased levels of inflammatory cytokines during the day rather than during the night [173,177]. These effects are observed after as little as one night of partial sleep loss [182,183,184,185]. Repeated or persistent periods of sleep loss/disturbance can lead to excessive and sustained activation of this inflammatory response, leading to chronically elevated systemic inflammation [186,187,188], as well as a host of other health-related problems underpinned by an inflammatory imbalance [189]. Like stress, these relationships are bidirectional and potentially cyclical—changes in the peripheral inflammatory status (e.g., after injury) can alter sleep via various mechanisms (for review, see references [173,190]).

Other lifestyle factors also impact systemic inflammation. For instance, physical activity/exercise strongly promotes anti-inflammatory processes and suppresses proinflammatory processes [191,192], whereas inactivity has the opposite effects [193,194,195]. Persistent physical inactivity leads to visceral adipose accumulation [196], which is a potent source of inflammatory factors [197], and is commonly accompanied by fatigue and muscle wasting—closing the negative cycle of inactivity, inflammation and poor health. Alcohol is another well-known immune modulator [198]. It can induce inflammation via agents derived from alcohol damaged cells and those derived from gut microflora, specifically LPS [121,199,200]. With respect to the latter, heavy alcohol consumption increases gut permeability and can cause leakage of gut microflora-derived agents (e.g., LPS) into the circulation and initiate an immune response [200]. Further, many foods, nutrients and non-nutrient food components modulate inflammation both acutely and chronically [201,202] via various mechanisms (for a review, see reference [203]), including those linked with increased adiposity and gut permeability. Cigarette smoke is another powerful modulator of inflammation, with far-reaching and complex effects on immunity that lead to constitutive inflammation, the skewing of adaptive T-cell-mediated immunity and impaired responses to pathogens, among others [204]. Encouragingly, most of these inflammatory-related changes are reversible.

### 4.3. Reconsidering Health of Musculoskeletal Connective Tissues from a Bio-Psycho-Social and Lifestyle Perspective

The hypothesis that the systemic inflammatory environment can substantially influence musculoskeletal inflammation and fibrogenesis, and that various psychosocial and lifestyle factors can impact systemic inflammation, provides a new understanding of the bio-psycho-social nature of musculoskeletal conditions and the need to consider musculoskeletal health from a “whole system” perspective that integrates both body and mind. Many of the psychological, lifestyle and biological mediators/moderators of systemic inflammation are reciprocally related, meaning a disturbance to any could have pathological consequences on the other, setting up a vicious cycle that results in excessive inflammation with potential implications for local tissues. On the flipside, this opens the enticing possibility that interventions aimed at one aspect (e.g., stress, sleep, physical activity or inflammation) could positively affect the others.

## 5. New Opportunities to Understand and Enhance the Effects of Treatments

An issue when viewing musculoskeletal health from a “whole system” perspective is that the list of potential contributors is vast. Although research has highlighted factors that potently impact inflammation (e.g., sleep, physical activity and stress/psychological factors), further work is necessary to understand the potential clinical relevance of these interactions for the development of connective tissue changes in musculoskeletal conditions. For instance, does the combination of specific factors have a cumulative—or greater—effect on inflammation and musculoskeletal health? Do “good” lifestyle choices (e.g., exercise) offset the impact of “bad” choices (e.g., poor sleep)? Do some factors have a greater short- and/or long-term impact on inflammation and tissue health when present earlier versus later in the injury cycle, and vice versa? The latter may have relevance for considering which factors to treat at different stages (e.g., acute, subacute or chronic) of the injury or condition. This information would be foundational for guiding treatments aimed at preventing and reversing these changes.

The hypothesis presented in this paper provides a foundation to consider potential roles for a diverse array of treatments for the prevention and resolution of connective tissue changes in musculoskeletal conditions. The new hypothesis suggests even the potential for tissue-level effects from interventions targeting psychosocial and lifestyle factors. Overall, the interaction between local and systemic inflammation highlighted in this hypothesis provides a foundation to consider the potential diverse effects of many interventions. For example, psychosocial interventions such as cognitive behavioral therapy (CBT) applied to address depression or insomnia can reduce systemic inflammatory activity [205]. Moreover, improvements in depressive symptoms and sleep are associated with reduced concentrations of circulating cytokines [206,207,208,209]. If the management of depression or sleep can reduce systemic inflammation, particularly TNF, then it is plausible that these inflammatory changes can also positively moderate local inflammatory processes via systemic-to-local pathways as described in Section 3. Exercise as an intervention can directly impact both systemic and local inflammation and, thus, has potential to impact both body and mind. Although the anti-inflammatory effects of exercise are largely systemic and attributed to changes in “non-muscle” factors/cells (e.g., reduced adipose tissue, macrophages, T- and NK cells and activation of the HPA axis) [210,211], the contracting muscle cells themselves produce IL-6, which inhibits TNF and stimulates the production of anti-inflammatory cytokines [212]—highlighting the dual systemic and local impact of exercise on inflammation, with potentially broad therapeutic implications. For instance, the potent anti-inflammatory effects of aerobic whole-body exercise [191,213] are accompanied by reduced musculoskeletal fibrosis [27] (unless injured tissues are loaded [214]) and improvements in psychological wellbeing and lifestyle health [215,216]. Other factors might have similar multidirectional effects. Many of the immune (e.g., inflammatory) and tissue changes associated with cigarette smoking and excessive alcohol consumption are substantially diminished, or even completely reversed, when reduced or ceased [217,218,219,220,221,222]. Again, these changes can positively impact both tissue- and higher- (psychosocial and lifestyle) level aspects [223,224]. Further, anti-inflammatory (e.g., systemic ibuprofen, anti-TNF and NSAIDs) treatments reduce not only systemic and tissue inflammation and fibrosis, but also markedly improve psychosocial features, including reversing/reducing clinical symptoms of depression and schizophrenia, and improving social interaction [80,84,104,225,226,227,228,229]. The underlying mechanisms are probably similar to those underpinning the psychological benefits of exercise, and likely include the anti-inflammatory “curbing” of proinflammatory alterations in the metabolism of neurotransmitters [150,151,230] (see Section 4.1). There is enormous potential to develop and refine targeted interventions that match the presenting confluence of factors that might drive the interaction between psychology/lifestyle—systemic and local inflammation—and fibrosis to preserve and restore the health of musculoskeletal tissues.

## 6. Conclusions

This paper presents a hypothesis that provides a new foundation to understand the connection between body and mind in musculoskeletal conditions. With the interaction between local and systemic inflammation at its core, the hypothesis provides a plausible and testable link from psychology and lifestyle to connective tissue health that might guide the development of more effective targeted treatments in musculoskeletal conditions.
